# Parvovirus B19 Infection due to over Immunosuppression in Kidney Transplant Recipients: Case Reports and Literature Review

**DOI:** 10.1155/2021/7651488

**Published:** 2021-11-29

**Authors:** Abdulrahman Altheaby, Malak Alotaibi, Nuha Alajlan, Ala Alshareef, Mohammed Alruwaymi, Ghaleb Aboalsamh, Mohammed F. Shaheen, Mohammed Alzunitan, Ziad Arabi

**Affiliations:** ^1^Organ Transplant Center and Hepatobiliary Sciences Department, King Saud bin Abdulaziz University for Health Sciences, King Abdulaziz Medical City, Riyadh, Saudi Arabia; ^2^Department of Medicine, King Saud bin Abdulaziz University for Health Science, King Abdulaziz Medical City, Riyadh, Saudi Arabia; ^3^College of Medicine, King Saud bin Abdulaziz University for Health Sciences, Riyadh, Saudi Arabia; ^4^Infection Prevention and Control Program, King Saud bin Abdulaziz University for Health Sciences, King Abdulaziz Medical City, Riyadh, Saudi Arabia

## Abstract

Parvovirus B19 (PB19) is a single-stranded DNA virus that belongs to the Erythrovirus genus within the *Parvoviridae* family. Clinical presentations associated with PB19 infection vary greatly, depending on the infected individual's age and hematologic and immunologic status. The limited data available regarding consensus on screening algorithms and indications in donors and recipients prior to kidney transplantation makes diagnosis and management challenging. We presented 3 cases of pure red cell aplasia due to parvovirus B19 after kidney transplant. These patients were diagnosed with severe normocytic, normochromic anemia (hemoglobin below 60 g/L) in the 1^st^ 6 months posttransplant. A complete anemia work-up revealed low reticulocyte count and was otherwise inconclusive. All patients were diagnosed with pure red cell aplasia due to parvovirus B19. Two patients improved after receiving intravenous immunoglobulin 2 gm/kg given over 4 doses. Unfortunately, they relapse after few weeks and required additional doses of intravenous immunoglobulin in conjugation with reduction of their immunosuppressive medication. The third patient improved after holding mycophenolate mofetil (MMF) and did not require intravenous immunoglobulin. Whereas PB19 infection is typically self-limiting and associated with positive IgM serology in immunocompetent hosts, these cases highlight the importance of considering PB19 infection in the differential diagnosis of persistent anemia in immunocompromised patients and the challenges in confirming the diagnosis. Intravenous immunoglobulin (IVIG) can be an effective treatment in immunocompromised patients with primary or relapsed PB19 infection in conjunction with minimizing immunosuppressive medication. Further research and consideration are required to determine appropriate and targeted screening in donors and recipients in the peritransplantation period.

## 1. Introduction

Parvovirus (Erythrovirus) B19 (PB19) is a common human infection worldwide. It belongs to the Erythrovirus genus within the *Parvoviridae* family [1]. PB19 transmission occurs by different routes that include respiratory transmission, vertically from mother to fetus, transfusion of blood or blood-derived products, or organ transplantation [2]. After PB19 infection, immunocompetent individuals are considered immune. However, reinfection has been suspected in some cases [2]. During active infection, the virus binds to globoside (P antigen) receptors that are present in erythroid precursor cells. The virus infects, replicates in, and then, lyses erythroid progenitor cells [2]. This will lead to pure red cell aplasia on bone marrow examination by revealing the presence of giant pronormoblasts which can help the diagnostic process [3].

## 2. Clinical Presentation

Clinical manifestations depend on host's hematological status and immune responses [1]. There is a wide spectrum of clinical findings in immunocompetent patients with PB19 infection. Most individuals who have serologic evidence of prior infection do not recall ever having any specific symptoms classically associated with PB19. The brief period of red cell aplasia is generally subclinical in otherwise healthy individuals, in children and adults with chronic hemolytic anemia (e.g., sickle cell disease or hereditary spherocytosis); the short-term pure red cell aplasia associated with PB19 infection leads to acute worsening of anemia, termed an “aplastic crisis,” often necessitating blood transfusion. In children, PB19 can manifest in erythema infectiosum, also known as the “fifth” disease, which presents as rash, fever, malaise, and polyarthropathy. In pregnancy, PB19 infection is associated with hydrops fetalis [4].

In solid organ transplant recipients, PB19 infection occurs on average 7 weeks (range, 1 week–8 years) after transplantation. Up to 65% of transplant recipients with PB19 infection present within the first 3 months following transplantation [5]. Anemia is an expected finding of this infection in solid organ transplantation. Fever presents in only 25% while arthralgia occurs in 6% of transplant recipients. Leucopenia and thrombocytopenia are seen in 33% and 18%, respectively, which is similar to noninfected transplant recipient's rates. Notably, skin rash is seen more frequently in hematopoietic stem cell transplant as opposed to solid organ transplant recipients (33% vs. 6%) [5]. Tissue-invasive disease was reported in 11% of transplant recipients, manifesting as hepatitis, myocarditis, pneumonitis, collapsing glomerulopathy, encephalitis, or vasculitis [6–9].

While not common, relapsing PB19-related anemia has been observed in 33% of patients treated with IVIG for primary PB19 infection. The risk of relapse is higher in patients with primary PB19 infection and patients who received antithymocyte globulin (ATG) for induction therapy [10].

## 3. Diagnosis

**Figure 1:**
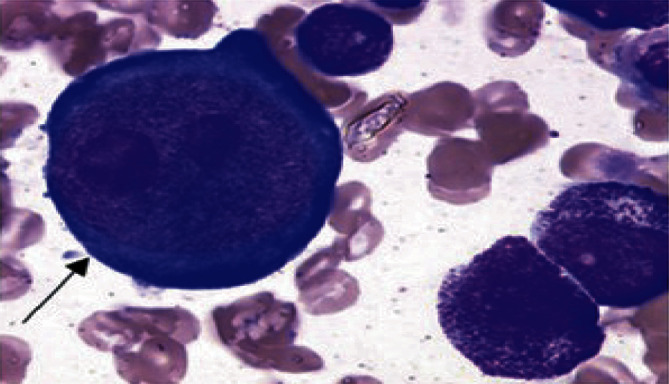
May–Grünwald–Giemsa staining of bone marrow aspirate showing the classical feature of PRCA, namely, a red inclusion in the nucleus of giant pronormoblasts (arrow) [11].

Diagnosis is based on the detection of PB19 IgG or IgM antibodies or PB19 DNA in blood or tissue samples by polymerase chain reaction (PCR). Bone marrow study may show giant pronormoblasts with prominent nuclear inclusions, which is characteristic of PB19 infection (Figure 1).

## 4. Management in Transplant Recipients

Management of presumed or confirmed PB19 infection in transplant recipients includes reduction of immunosuppressive medication. Some case reports have shown recovery after holding tacrolimus or substitution of tacrolimus with cyclosporine [12]. While intravenous immunoglobulin (IVIG) has also been used to treat PB19 infection in many transplant recipients, with successful resolution of viremia [5, 13, 14], some other patients experienced long-lasting resolution of the infection without receiving IVIG [5]. Although the optimal dose of IVIG and the frequency of administration are not well studied, a dose of 400 mg/kg/day for 5 days has been recommended in some studies [5].

## 5. Case Presentation

### 5.1. Case 1

A 43-year-old man had a deceased-donor renal transplantation for the management of end-stage renal disease secondary to IgA nephropathy. The induction agent used at the time of transplantation was ATG 4.5 mg/kg. Afterwards, he received immunosuppressive maintenance therapy that consisted of mycophenolate mofetil (MMF) 1000 mg twice daily, tacrolimus, and prednisone 5 mg daily. The donor's and recipient's PB19 statuses were unknown.

After the transplant, hemoglobin remained stable in the range of 100-120 g/L until 4-month posttransplantation when the patient started to complain of dizziness and shortness of breath on exertion. Blood work at that time showed hemoglobin level of 58 mg/L that was normocytic and normochromic with normal morphology on the blood smear. The patient's reticulocyte count was inappropriately low at 3 × 10^9^/L (normal range 10 − 86 × 10^9^/L). Vitamin B_12_ and iron indices were normal, and the hemolytic screen was negative. PCR for Cytomegalovirus (CMV), BK virus, and Epstein-Barr virus (EBV) was negative. Serology for PB19 showed a negative IgG and an indeterminate IgM antibody level. The patient was transfused 2 units of red blood cells and discharged home on erythropoietin stimulating agent. MMF dose also reduced to 500 mg twice daily upon discharge.

Unfortunately, the patient was readmitted 2 weeks later with the same complaints and a hemoglobin level of 56 g/L. The reticulocyte count was persistently low at 5 × 10^9^/L. PB19 serology was repeated and showed negative IgG and an indeterminate IgM. The patient received transfusion of 2 units of RBCs and PB19 PCR also positive this time. Patient started on IVIG (0.5 g/kg) for a total of 4 doses. MMF discontinued on this admission and patient discharge after completing IVIG course on prednisolone and tacrolimus only. On discharge, the patient's symptoms had improved, and hemoglobin was 87 g/L. Patients followed monthly in posttransplant clinic, and hemoglobin normalized after 1 month. MMF resumed at 500 mg bid after 4 months, and hemoglobin remained above 125 g/L during 3 years follow-up with no further relapse.

### 5.2. Case 2

A 26-year-old female had a deceased-donor renal transplantation for the management of end-stage renal disease secondary to Alport syndrome. The induction agent used at the time of transplantation was ATG 4.2 mg/kg. Afterwards, she received immunosuppressive maintenance therapy that consisted of MMF 1000 mg BID, Tacrolimus, and prednisone 5 mg daily. The donor's and recipient's PB19 statuses were unknown.

The patient developed severe symptomatic, normocytic, and normochromic anemia (hemoglobin 55 g/L) 6 months after transplantation with normal platelet and white cell count. A complete anemia work-up showed low reticulocyte count; otherwise, inconclusive. Her severe anemia persisted for over 2 weeks despite packed blood cell transfusion, adjustment of immunosuppressant medications, and administration of erythropoietin stimulating agent. PB19 PCR was sent few days after admission and came back positive. MMF was held, and 2 gm/kg of IVIG was given over 4 doses. She was discharged few days after completing the IVIG course with better hemoglobin (90 g/L). The patient was seen after 2 weeks in the clinic with almost normalized hemoglobin (124 g/L). Hence, MMF 500 mg twice daily was restarted. 5 weeks later, the patient presented again to the emergency department with fatigability and shortness of breath and a hemoglobin level of 52 g/L. Another course of IVIG treatment was initiated. She received a total of 2 gm/kg over 4 doses, and MMF was held again. PB19 PCR came back positive yet again. After treatment, she improved and was discharged home. The hemoglobin level normalized after few weeks. Hence, MMF 500 mg BID was restarted. For the third time, the patient represented with severe anemia requiring blood transfusion. She was given IVIG 2 gm/kg again and discharge with a plan to discontinue MMF permanently. Her hemoglobin remained stable during the subsequent 2 years of follow-up with no further relapse.

### 5.3. Case 3

A 24-year-old female had a combined diseased donor kidney and liver transplant. The induction agent used at the time of transplantation was ATG 4 mg/kg. Afterwards, she received immunosuppressive maintenance therapy that consisted of MMF 1000 mg BID, Tacrolimus, and prednisone 5 mg daily. The donor's and recipient's PB19 statuses were unknown.

One month after discharge, the patient presented to the clinic with a hemoglobin drop reaching 61 g/L. The anemia was normocytic and normochromic with low reticulocyte. A complete anemia workup was inconclusive. The patient was already on an erythropoietin stimulating agent. PB19 PCR test was sent and came back positive. Patient was also found to have BK viremia with 11000 copies detected via PCR. MMF was discontinued, and she was followed in transplant clinic weekly. Her hemoglobin improved and reached 123 g/L after 4 weeks and she did not require IVIG. BK viremia resolved after 2 months as well. Her hemoglobin remains stable during the subsequent 2-year follow-up period. The MMF was never resumed.

## 6. Discussion

Kidney transplant recipients are highly susceptible to infections such as PB19 [15]. This is due to the use of potent immunosuppressive agents such as ATG to prevent early acute rejection and because of the effect of sustained long-term immunosuppression to prevent graft loss. In this case series, we are presenting 3 cases presented with symptomatic, normocytic, and normochromic anemia with normal platelet and white cell count. They have positive PB19 PCR. Although posttransplant anemia could be related to medication such as ATG and MMF, this is unlikely in our cases because these medications mainly cause leucopenia and thrombocytopenia as well, in addition to positive PB19 PCR in our cases that suggest pure red cell aplasia due to PB19 infection.

PB19 infection in transplanted patients often leads to pure red cell aplasia by attacking erythroid progenitor cells in the bone marrow [16]. Induction with ATG was found to have a higher risk for PB19 infection compared to the other induction agents [17]. Maintenance regimen also plays a role in the risk for PB19 infection as one study showed substitution of tacrolimus with cyclosporine was followed by resolution of anemia and viral clearance [11]. The presence of viral coinfection with PB19 such as CMV and human herpesvirus 6 has also been reported. In our case series, the noted patients' risk factors for PB19 infection and relapse included induction with ATG and having negative IgG on PB19 serology testing suggesting a primary infection, and not reactivation. Similar to other case reports, one of our patients also has a coinfection with BK virus [18].

PB19 infection is likely underdiagnosed in organ transplant recipients, and a high index of suspicion is necessary to allow for prompt identification of PB19 infection among renal transplant recipients with anemia of unclear etiology.

Our case series further suggests that the treatment of PB19 relies on the reduction of immunosuppression and IVIG administration. There is no clear evidence to support the best practice for adjusting the immunosuppressive medication. Some case reports showed recovery after holding tacrolimus or substitution of tacrolimus with cyclosporine [11]. In our cases, we discontinue MMF in all 3 cases by the end. These cases suggest that treatment with IVIG is effective in treating primary infection and relapse, but holding antimetabolite immunosuppressive drugs is essential in treating such cases and preventing further relapses, although we do not know when should we resume it.

## 7. Conclusion

Whereas PB19 infection is typically self-limiting and associated with positive IgM serology in immunocompetent hosts, these cases highlight the importance of considering PB19 infection in the differential diagnosis of persistent anemia in immunocompromised patients and the challenges in establishing the diagnosis. IVIG may be an effective treatment in immunocompromised patients with primary and relapsing PB19 infection. Minimizing or holding antimetabolite immunosuppressive drugs plays a vital role in treating and preventing PB19 relapse. Further research and consideration are required to determine an appropriate screening protocol for donors and recipients in the peritransplantation period.

## Data Availability

The detailed case data used to support the findings are available from the corresponding author upon request.
